# Actions of primary health care professionals to reduce maternal mortality in the Brazilian Northeast

**DOI:** 10.1186/s12939-018-0817-x

**Published:** 2018-07-16

**Authors:** Katia M. S. Figueiredo, Gleice A. A. Gonçalves, Hermes M. T. Batista, Marco Akerman, Woneska R. Pinheiro, Vânia B. Nascimento

**Affiliations:** 1 University Center Dr. LeãoSampaio – UNILEÃO, Rua Maria Cornélio de Lira, n° 26, CEP: 63022-310, bairroAntônio Vieira, Juazeiro do Norte, CE Brazil; 2 University Regional of Cariri – URCA, Crato, Brazil; 3 Anesthesiologist, Hospital Regional of Cariri, Juazeiro do Norte, Brazil; 4 University of São Paulo – USP, São Paulo, Brazil; 5 College of Medicine ABC – FMABC, Santo André, Brazil

**Keywords:** Maternal mortality, Comprehensiveness, Public policy

## Abstract

**Background:**

Maternal mortality is a global public health problem. Statistics show that in 2013, 289,000 women died from complications during pregnancy, childbirth or the postpartum period worldwide. Between 2010 and 2015, there were 10,075 maternal deaths in Brazil, 3,522of which occurred in the Northeast region. The aim of this study was to investigate the actions taken by primary health care (PHC) professionals to reduce maternal mortality.

**Method:**

This was a cross-sectional, descriptive field study with a qualitative approach. The sample comprised 81 graduate-level professionals working in PHC in the state of Ceará, Brazil. Data were collected from January to March 2016 using structured interviews, which were digitally audio recorded and transcribed. The results were organized using collective subject discourse and analyzed according with the relevant literature.

**Results:**

The PHC professionals took both individual and joint measures to reduce maternal mortality. These activities included home visits, health education, active searches, prenatal care consultations, referrals to specialized care and outreach. The challenges that must be overcome to prevent maternal mortality include poor care and ineffective public policies that are associated with a lack of managerial support.

**Conclusion:**

Interaction among professionals in the health care network is critical to the development of cross-sectoral projects that improve the quality of women’s health care. Prenatal care is a key factor in reducing maternal death and enables the identification and classification of the risks to which pregnant women may be exposed and the implementation of early actions that can ensure a safe and uncomplicated delivery. However, all of these actions require effective public policies and managerial support.

## Background

Maternal mortality rates have been high for many years. At the beginning of this century, representatives of 189 countries met and created a set of quantifiable objectives and targets known as the Millennium Development Goals, and they stipulated deadlines for achieving these goals. The fifth goal, “improve maternal health”, proposed a 75% reduction in maternal mortality within 15 years (2000–2015) [1]. This goal was not achieved, especially in developing countries.

The World Health Organization considers a maternal mortality rate (MMR) of 20 maternal deaths per 100,000 live births acceptable. The MMR is calculated by multiplying the total number of maternal deaths in a given period by 100,000 and dividing that number by the total number of live births in the same period [2].

The MMR data for 2013 reveal the following: in South Asia and sub-Saharan Africa, there were 510 deaths/100,000 live births; in Latin America, 77/100,000; in Ireland, 9/100,000; in Canada, 11/100,000; in Afghanistan, 400/100,000; in Haiti, 380/100,000; and in Brazil, 69/100,000 [3]. These data demonstrate that developing countries have a higher MMR than developed countries.

According to a public database, from 2010 to 2015, Brazil had an MMR of 57.3/100,000 live births. The Southeast region had an MMR of 54.5/100,000 live births, while the Northeast region had an MMR of 70/100,000 live births [4]. Residents of the Northeast region have access to three levels of health care: primary, secondary and tertiary. Nonetheless, maternal death rates in that region are well above those recommended by the World Health Organization.

In Brazil, public health needs are met by a unified health system (UHS), which aims to provide free and public care for all citizens regardless of their social, demographic or ethnic characteristics. This system was instituted in 1990 with the creation of Organic Health Law No. 8088, which established conditions for the promotion, protection and recovery of health and defined the organization and functions of the corresponding services [5].

The UHS is organized according to doctrinal and organizational principles. Its doctrinal principles are universality, equity and comprehensiveness; its organizational principles are regionalization, hierarchy, resolvability, decentralization, user participation and cooperation with the private sector [6].

The UHS is part of the Brazilian social security system. Social security guarantees health, welfare and social assistance and prioritizes universal coverage and treatment for the entire population. Its health services integrate a regionalized and hierarchical network organized at each level of government (municipal, state and federal) according to the principles of decentralization and integrated care. Such actions and services promote curative and preventive measures and ensure community participation through health councils and conferences [7].

The UHS has the responsibility for resolving public health issues, and reducing maternal mortality is one challenge that the system faces. Thus, in 2011, the Brazilian government adopted strategies to reduce maternal and infant mortality rates by means of Ordinance No. 1459, the Mother and Child Network, known as the Stork Network. This network provides care designed to ensure the rights of women, such as reproductive planning and humanized care during pregnancy, childbirth and the postpartum period, and to protect the child’s right to a safe birth and healthy growth and development [8].

The Stork Network is part of the health care network and provides support to primary health care (PHC) services. It is the preferred entry portal for prenatal health care services, and it connects pregnant woman with the maternity hospital responsible for their childbirth. The portal is also responsible for postpartum care and for providing comprehensive health care for the child [8].

In the UHS, women’s PHC is the responsibility of the Family Health Strategy (FHE) and the Support Center for Family Health (SCFH). The FHE was established in 1994 to restructure the UHS and move it away from the hospital-centered model of health care. Its aim is to make health more accessible to families to improve the population’s quality of life. The SCFH was introduced in 2008 by Decree No. 154 to support the inclusion of the FHE in the service network and expand its range, resolvability, territorialization and regionalization [9].

According to reports available in a public database, 80.40% of the population of the Northeast region was covered by the FHE at the end of 2017, and 82.5% of the population in the state of Ceará was covered during the same period. These data reflect the monitoring of access to PHC services and the strengthened planning of actions undertaken at this level of care [10].

It is important to associate the prevention of maternal mortality with social determinants of health, such as income, education level, unplanned pregnancy and abortion, which are among the main determinants of a higher MMR. These issues should be analyzed in the context of the maternal health-disease process.

In Brazil, abortion is legal in only a few situations: when it is the only way to save the life of the pregnant woman, when the pregnancy is the result of rape or incest, and (in some cases) when the fetus has a congenital malformation [11]. However, studies show that abortion in Brazil is widely used under circumstances that are not legally sanctioned, and many women resort to induced abortion as output to unplanned pregnancy. Abortion is therefore a major cause of maternal mortality [12].

In this context, the questions guiding this study were based on the acronym PICO: P –population (i.e., PHC professionals), I –intervention (i.e., actions taken to reduce maternal mortality and related challenges), C –comparison (i.e., MMR), and O –outcome (i.e., reducing maternal mortality). As a result, the following questions were raised: What actions do PHC practitioners take to reduce maternal mortality? What types of training and skill development are available to them? What challenges do they face in reducing maternal mortality?

Given the high MMR in Brazil, particularly northeastern Brazil, it is important to address these questions to promote pregnant women’s health and prevent mortality during pregnancy and childbirth. The aim of this study was to investigate the actions performed by PHC professionals and their capacity, aptitude and challenges related to reducing maternal mortality.

## Methods

This was a cross-sectional and descriptive field study with a qualitative approach. It was performed in southern Ceará, a northeastern Brazilian state. The purposeful sample comprised graduate professionals working in the FHE and SCFH, including physicians, nurses, dentists, social workers, physical educators, pharmacists, physiotherapists and psychologists.

Northeastern Brazil has a warm climate for much of the year. It is the third largest region in Brazil, with a territory of approximately 1,558,196 km^2^, and it comprises nine states. It has a population of approximately 53 million inhabitants and a population density of 32 in habitants/km^2^. Its Human Development Index is 0.659, which is considered average. The gross domestic products BRL 848,532 billion, which represents 14.2% of the gross national product [13].

Data collection took place from January to March 2016 in a municipality with 70 FHE teams and 7 SCFH teams, for a total of approximately 240 professionals. The study included 81 of these professionals; 42 declined to participate, and the rest did not meet the inclusion criteria described below. Among the professionals who participated, 6 did not want or know how to respond regarding their actions to reduce maternal mortality, and 4 did not indicate what challenges they faced in doing so. The professionals in the sample met the following inclusion criteria: a) having worked on an FHE or SCFH team for at least six months, b) working on an FHE team that engaged in SCFH activity, c) performing usual-risk prenatal care and, for mothers who met the criteria for high or medium risk, providing coordinated care between the PHC and a specialized service, and d) fully exercising their activities during the data collection period.

A structured form was used to collect data during interviews. The interviews were audio recorded digitally and transcribed to allow the researcher flexibility in obtaining additional important information to meet the proposed objectives.

To verify that the questions contained in the form were significantly likely to meet the study objectives, a pre-test that included 5 randomly chosen PHC professionals who volunteered to participate was carried out. After reading each item, the professionals were asked to describe their understanding of what was being asked in their own words. When the purpose of the question and the professionals’ understanding was consistent, the item was kept on the form; if they diverged, the item was reformulated according to the professionals recommendations. To evaluate the content of the form, two experts in women’s health and maternal mortality were consulted. After the experts’ analysis, 5 open questions that met the study’s proposed objectives remained on the form.

Data collection occurred during the work hours of the FHE professionals. First, each professional was contacted to explain the purpose of the research and to confirm his/her availability to participate in the study. If the professional was available at this point, the interview was conducted; otherwise, it was scheduled according to the participant’s availability.

The data collected in the interviews were transcribed in full and organized using the collective subject discourse method, in which central ideas/anchors and their corresponding key expressions were obtained. This method consists of obtaining the central ideas and their corresponding key expressions,which make up one or several speech syntheses. Speech syntheses are the discourses of the collective subject, which in this case refer to the terms, actions and challenges that the professionals identified in association with efforts to reduce MMR.

In the social representation created via collective subject discourse, individuals’ identities are transmuted, dissolved and incorporated into one or several collective discourses [14].

Collective subject discourse is a discursive data organization technique used in qualitative research that enables a community to speak as if it were a single individual. The raw material used in collective subject discourse comprises thoughts expressed in discursive form. The discourses are subjected to content analysis, which begins by decomposing each discourse into its main central ideas and determining the main central ideas of the discourses as a whole; a summary that reconstitutes the discourse of the group is then created [14].

After the data were organized using collective subject discourse technique, three themes and their respective central ideas emerged. Theme 1 was the actions that PHC professionals took to reduce maternal mortality; its central ideas were home visits, health education, active searches, consultations, referral to specialized care and outreach. Theme 2 was professional competence; its main ideas were training and skill development to reduce maternal mortality. Theme 3 was challenges in reducing maternal mortality; its central idea was effective public policies.

In compliance with Resolution No. 466/12 of the National Health Committee (Comitê Nacional de Saúde) [15], the study was submitted to the Research Ethics Committee of the University Center Dr. Leão Sampaio, record No. 5048, and was approved on January 15, 2016, under protocol No. 1.389.345.

## Results

The results were organized into the three above mentioned themes, with their respective central ideas and collective subject discourse. The discourse analysis was based on the participants’ reports of the individual and joint measures they took to reduce MMR, the challenges they faced in carrying out those actions and the skill development opportunities that were available to them to reduce MMR.

### Actions taken by primary health care professionals to reduce maternal mortality

**Table:** 

Central ideas: Home visits, health education, active searches, consultations, referral to specialized care and outreach.	
“I conduct home visits, health education and individual care. At this point, I raise the issue of rights, health promotion, hygiene and especially the importance of prenatal care and family planning counseling before pregnancy. The main strategy in promoting the health of pregnant women involves the active pursuit of these patients in the community, bringing them in for prenatal consultation. [Responsibility for this strategy] is shared: one month with the doctor and the other with the nurse. We warn them [the mothers] about the importance of this monitoring for the pregnancy to go smoothly and provide guidance on risk factors. We also ask for tests to be performed, and if some problem or risk is identified that might affect the pregnancy or the woman’s health in general, we send the pregnant woman to a specialist service. I see the nurse as the main professional responsible for the prenatal care of this population. The nurse also coordinates outreach to groups of these women.”	

The aim of this study was to investigate how actions directed toward women’s health care are carried out in an attempt to reduce maternal mortality. In this context, the speech synthesis revealed that home visits, health education, active searches, consultations, referral to specialized care and outreach are tools for reducing maternal mortality.

### Professional competence

**Table:** 

Central ideas: Training and skill development to reduce MMR.	
“I have confidence in carrying out my work while I am active in PHC. There are actions and tools that can be used to reduce this problem. In my area of expertise, I have this training and recognize the importance of acting in conjunction with other professionals in the team. I notice a lack of closeness between FHE and SCFH professionals and between professionals working in primary, secondary and tertiary care. I emphasize the need for training on the subject, so we can act in a comprehensive way and perform this assignment better because everything that is done by each professional area of expertise can benefit pregnant women, and the more knowledge you have of a particular subject, the more power you will have to act and try to change reality. There is also the matter of working in isolation from one another and lacking proper conditions in terms of infrastructure, equipment and tests. If we had better conditions, then our actions would be more concrete.”	

The PHC professionals indicated that teamwork is important to their ability to act to reduce MMR. The speech synthesis revealed that the professionals have confidence in the activities they perform and emphasized the importance of working as a team. The analysis indicated that each professional is important in the provision of holistic care and that working individually is not conducive to satisfactory health promotion or the prevention of maternal mortality.

### Challenges for reducing maternal mortality

**Table:** 

Central idea: Effective public policies	
“It would be the effectiveness of public health policies, as well as showing the professionals working at the point of care who have a broader view, noting other care dimensions for this audience, performing our actions and providing early identification of women who deserve specialized care, referring them when necessary, so that everyone, including management, demonstrates a commitment to this fight. There must be management commitment to providing better working conditions, so professionals can act appropriately– both in PHC and at medium- and high-complexity levels of care – with the necessary resources to provide the community with optimized and with more quality healthcare coverage, ensuring that pregnant women are treated in a comprehensive way and feel confident in using the health service, and in return we can offer care, guidance and support.”	

The speech synthesis suggested possible measures to reduce maternal mortality. In particular, it highlighted the effectiveness of public policies that promote comprehensive women’s health care, and it focused on the commitment of management in PHC and care at medium- and high-complexity levels of care.

## Discussion

Health education promotion was one of the actions taken by PHC to reduce maternal mortality. However, ascertaining the education level of the treated woman is necessary, as familiarization with the educational level of the target audience is essential when planning activities to provide knowledge and increase awareness about a particular issue.

Pregnant women’s education level is an important factor to consider in the context of health care. In general, women with higher education levels tend to seek more preventive care, which contributes to a substantial reduction in maternal mortality levels [16].

Women’s education levels can significantly affect their maternal health. Consequently, education is a social determinant of health that may enhance strategies for improving individual health because the higher the population’s education level is, the greater its demand for social and health rights that make equal opportunities available for everyone [17].

Studies indicate that the causes of maternal mortality are not equally distributed and this inequality is attributable not only to deficiencies in the health system but to social determinants of health. Thus, the risk of maternal mortality is higher in socially disadvantaged populations since women in these circumstances are more likely to experience unintended pregnancy than women with greater financial and social resources [18, 19].

Education, income and occupation are closely related and can determine access to assets and social opportunities; consequently, they assume an important role in the success of health actions such as reproductive planning and reducing maternal mortality. Financial dependence and inadequate income can also be determinants of unplanned pregnancy and unsafe abortion practices.

In Brazil, abortion is among the five main causes of maternal mortality, and it is related to approximately 5% of all maternal deaths. For this reason, in recent years, there has been a great debate in the country regarding the decriminalization of abortion, which involves a complex set of political, legal, moral, religious, social and cultural aspects [20].

According to data from the World Health Organization, Brazil is in the lead in the number of induced abortions, with a total of four million per year. One in nine Brazilian women uses abortion as a means of ending an unplanned pregnancy. However, it is not known precisely how many women have abortions or how many die annually from abortion-related causes because, as abortion is illegal in Brazil, it occurs predominantly under clandestine and precarious conditions; such circumstances contribute to the role of abortion in the high maternal mortality numbers in our country [21].

Women residing in suburban areas and rural communities are more susceptible to maternal death. Age is also a factor; maternal death is more common in women under 15 and over 35 years old. Regarding education, illiterate women and those with a low education level are more susceptible to maternal death. In Brazil, because of racial miscegenation, it is difficult to associate race with the maternal death rate, but a 2006 study found that the MMR was higher among white women (61.5%) than among black women (17.9%). Regarding marital status, single women are more vulnerable to maternal death, and abandonment has been identified as a contributing factor [18, 22].

Among the strategies for promoting health and reducing maternal mortality, health education can have a positive effect by reducing health inequities and promoting the autonomy and empowerment of individuals and social groups [23]. Thus, health education is an important factor to consider for reducing health risks. Education is also relevant to the multidisciplinary and interdisciplinary work of PHC professionals because it allows them to detect and analyze several variables, including social determinants of health, that are potential maternal health risk factors.

Home visits were cited as a health promotion strategy for pregnant women. When conducted during the postpartum period, such visits are an efficient way for professionals to monitor the prenatal and postpartum periods and interact with the family. These visits provide an opportunity for the woman to show the professional her actual living conditions, and they allow the professional to observe the family and social context that the woman operates, which can facilitate genuine and humanized actions [24].

In this context, monitoring the postpartum period at the woman’s home facilitates safety and allows the professional and the family to identify factors that may put the health of the mother or child at risk. Once the family is able to identify risks to maternal health, it may request PHC support and take measures to reduce maternal death.

Active searches were also identified as an action that health care teams performed within the community to bring pregnant women for prenatal care. Studies [25, 26] show that active searches are a proactive way for PHC professionals to address the health problems of the population they treat. Active searches strengthen the reorganization of PHC professionals’ work process and consequently encourage prenatal care.

Another action that the study participants highlighted was prenatal consultations. Prenatal consultations provide an ideal opportunity to identify any risks to which pregnant women may be exposed. Risks can be identified via physical examinations, laboratory tests and imaging. When the professionals observed risks to the health of amother and/or child, they could contact the health care network to provide interventions. When they referred a pregnant woman to another level of health care, they were seeking comprehensive care for the woman. Access to specialized services occurs through referrals and is aimed at the pregnant woman’s well-being. Such access can minimize maternal health consequences, especially for women experiencing pregnancy-associated risk factors such as hypertension, obesity and gestational diabetes mellitus [27].

The PHC system emphasizes humanized attention to the woman during the pregnancy cycle. The professionals participating in this study pointed out the importance of a humanized approach that provides an appropriate welcome, qualified listening, appreciation of the demands on the pregnant woman and respect for her privacy. This approach supports professionals’ ability to interact with women and identify social, demographic and health risks that may threaten their lives. This provides an opportunity for health services to act early, avoiding a disastrous outcome such as death [27].

Professional competence, which reflects the PHC professional’s ability to perform their work with a recognition of the morbidity and mortality profile of the population being served, is an important component of the integration of health actions; it is possible to assess professionals’ performance according to the health needs of the population [28]. By identifying risks to the health of pregnant women, it should be possible to involve other professionals and other segments of the health care network in their care. The ability to act as a team is directly related to organizational work processes and allows professionals to interact and develop care plans that promote the integration of professionals working in PHC [29].

This study shows that for professionals to be skilled and fully equipped to recognize and address gestational risk factors, they should receive training, which can be accomplished through the lifelong learning policy. This policy, which includes pedagogical concepts related to teaching health workers about management and social control, aims to support work processes related to planning and organization [30].

In the context of providing adequate maternal health services, professionals face factors related to infrastructure and facilities, resource availability, training, professional promotion and knowledge of the reality and characteristics of the population they serve [31]. The precarious infrastructure, the dearth of public services and a lack of medicine, tests and supplies may affect professionals’ ability to provide effective care.

The study participants perceived a need for the FHE and SCFH to work together more closely in cross-sectoral efforts. The proposal of cross-sectoral work is related to the development of shared and integrated actions with the aim of sharing knowledge and experiences in a way that has a positive effect on individuals’ or communities’ living conditions [32].

Therefore, to advance the development of cross-sectoral projects, it is essential to facilitate a commitment to health promotion actions and to improving people’s quality of life [33]. To carry out health promotion actions, it is necessary to connect the health sector to other public policy sectors, particularly education, public safety, city infrastructure and social policies.

It can be said that health care network integration ensures the equity of health services and the satisfaction and quality of life of users and their families as it promotes the rational use of health services and minimizes financial burdens [34]. However, integration occurs insufficiently, which presents a major challenge in the provision of health care.

In discussing the challenges of reducing MMR, the PHC professionals identified four attributes that are essential to health care for women during pregnancy and child birth: Access to care, continuity of care, comprehensiveness of care and coordination of care within the system. The Ministry of Health outlines that management must provide infrastructure, equipment and supplies at all health care levels and must promote lifelong learning to ensure the comprehensiveness of health care [35].

Studies indicate that a reduction in the MMR is associated with expanded PHC coverage for the population by means of FHE teams. The decentralization of health services offers improved access and thus can increase the availability of maternal and child health care, curative and preventive care, health promotion, home visits and referrals [35].

Effective public policies to ensure the quality of care represent a key factor in preventing maternal death. However, the effectiveness of public policy is independent of factors related to professional qualifications and available technological resources. In this context, public health policies should aim to identify the social determinants of health and the needs and rights of vulnerable individuals and groups, promote the comprehensiveness of and access to health systems, and eliminate discrimination and inequity [36].

It is the responsibility of the public administration to implement policies that are efficient, immediate and involve all sectors of the health care network. This will enable professionals in the health services to respond in a timely manner to demands to perform a simple procedure or secure an inpatient intensive care unit to provide adequate treatment. It is also important to staff health care services with professionals capable of intervening in cases that require specific attention. These conditions are considered essential for reducing MMR.

### Study limitations

The study has limitations. The first is the discontinuation of the Federal Mortality Information System, which at the time of the study provided epidemiological data on maternal mortality up to 2015. In addition, the professionals comprising the sample did not always respond to all interview questions.

## Conclusions

Maternal and child health care is regulated by public policies, and it is a government priority to reorganize and strengthen women’s health care and reduce MMR. Reducing these rates is contingent on certain factors, and as this study shows, measures related to professional training, municipal management, and the pregnant woman and her family are integral to the fight against maternal mortality.

The study shows that interdisciplinarity among PHC professionals facilitates integrated care for pregnant women. Actions that reduce the MMR include outreach, prenatal monitoring consultations, home visits, health education, active searches, and referrals to specialized care.

One challenge in reducing maternal mortality is the discrepancy between what public health policies advocate and the applicability of those policies in practice. This study revealed a lack of integration of these policies. Their ineffectiveness could be correlated with the lack of the necessary infrastructure and basic resources to provide care for pregnant women in PHC.

## Abbreviations

FHE: Family Health Strategy
MMR: Maternal Mortality Rate
PHC: Primary Health Care
SCFH: Family Health Support Center
UHS: Unified Health Care
